# Screening of organic substrates for the development of effective biofungicides to manage cashew fusarium wilt disease

**DOI:** 10.14440/jbm.2025.0089

**Published:** 2025-02-21

**Authors:** Stanslaus A. Lilai, Juma Hussein, Fortunus A. Kapinga, Wilson A. Nene, Stela G. Temu, Donatha D. Tibuhwa

**Affiliations:** 1 Department of Research and Innovation-Cashew Research Programme (CRP), Tanzania Agricultural Research Institute, Naliendele, Mtwara 63115, Tanzania; 2 Department of Molecular Biology and Biotechnology, College of Natural and Applied Sciences, University of Dar es Salaam, Dar es Salaam 16103, Tanzania

**Keywords:** Agro-wastes, Grains, Biological control agents, Fermentation media, Shelf life

## Abstract

**Background:**

The biotechnology industry commonly utilizes synthetic media to grow biological control agents (BCAs); however, these media are often considered impractical, particularly in developing countries.

**Objective:**

This study aimed to identify the most suitable locally available organic substrates for the cultivation of BCAs used against cashew fusarium wilt disease.

**Materials and Methods:**

Experiments were conducted in 2022 and 2023 on five substrates, namely rice husk (RH), rice bran (RB), hulled millet, parboiled rice, and their combinations, as growth media for combined *Bacillus* strains and *Trichoderma asperellum*. The carbohydrate and protein content of the substrates were quantified colorimetrically.

**Results:**

Protein and carbohydrate contents ranged from 13.4 – 19.8% to 76.3 – 82.7%, respectively. The BCAs exhibited high colonization rates on all tested substrates, with combined substrates supporting the highest colonization, followed by RH and RB. Endospore formation and conidiation levels varied significantly over time across substrates and incubation temperatures (*p* ≤ 0.05). Population densities decreased over time under refrigerated, room temperature, and incubator conditions for most substrates in both seasons, except for combined substrates, RH, and RB. Final population counts were as follows: 2.1 × 10^7^ ± 4.9 × 10^5^ and 1.0 × 10^7^ ± 0.3 × 10^5^ colony-forming units (CFUs)/mL (combined substrates), 2.3 × 10^7^ ± 5.4 × 10^5^ and 5.7 × 10^7^ ± 1.1 × 10^6^ CFUs/mL (RH), 3.6 × 10^5^ ± 2.1 × 10^4^ and 3.3 × 10^5^ ± 1.6 × 10^4^ CFUs/mL (RB), while under refrigerated conditions, population densities remained relatively unchanged throughout the study period.

**Conclusion:**

Based on the findings, it is recommended to use a mixture of substrates, RH alone, or RB alone as appropriate media for the large-scale production of the studied biofungicides.

## 1. Introduction

Synthetic media are commonly used in laboratories and the biotechnology industry for the cultivation of biological control agents (BCAs).^1^ Effective BCAs must exhibit pathogen suppression, environmental tolerance, prolonged shelf life, cost-effectiveness, and rapid proliferation in the rhizosphere.^2^ However, the reliance on synthetic media for the production of BCAs presents several challenges and limitations, such as high costs, limited accessibility, and availability, compared to locally available organic substrates.^2^ Thus, adequate availability of nutrients, including proteins, carbon sources, vitamins, and mineral elements, as well as optimal temperature, pH, and storage conditions is crucial for selecting suitable locally available organic substrates for cultivating appropriate biocontrol microbes and developing organic-based substrate bioformulations.^3^ The use of inexpensive, locally available agricultural wastes and cereal grains, offers a viable alternative to synthetic growth media for biocontrol microbes. Various substrates, such as rice husks (RH), rice grains, wheat bran, sorghum, maize oil cakes, and cereal straws, have proven effective for the proliferation of microorganisms due to their nutritional composition.^4^-^7^

The industrial production of biofungicides has been conducted using two methods: solid-state fermentation (SSF) and liquid-state or submerged fermentation (SmF).^2^ Most filamentous fungi (molds) are cultivated under SSF rather than SmF because SSF involves the cultivation of BCAs on an organic solid substrate, which offers numerous advantages. These benefits include providing structural support, lower water activity, higher fermentation productivity, enhanced product stability, and better mimicry of natural microbial growth environments.^8^ In contrast, bacterial genera are typically cultivated using SmF due to their higher water activity requirements and the need for even nutrient distribution within the growing media.^1^ Utilizing low-cost, locally sourced agricultural wastes and cereal grains provides an effective substitute for synthetic media for microbial growth.^9^ In addition, key aspects of bioformulation include the development of innovative methods to optimize the quantity, quality, and shelf-life of microbial products.^10^ For example, in crop protection, the success of a biofungicide depends on several factors, including costs, application methods, appropriate storage (packaging) materials, and the nutritional content of the growing media. The selection of appropriate substrates plays a critical role in promoting microbial growth, with various materials offering advantages due to their rich nutritional composition,^11^,^12^ including parboiled millet, wheat bran, wheat husks, sawdust, banana husks, and bagasse.^4^

Biological control is considered one of the most effective alternatives for managing plant diseases, as it is sustainable and environmentally safe.^13^,^14^ Studies have shown that certain members of the genera *Bacillus* and *Trichoderma* are among the best biocontrol candidates against numerous soilborne phytopathogens.^15^-^17^ However, chemical pesticides are still widely used, raising concerns about their adverse environmental effects. These effects include altering microbiomes, fostering pathogen resistance, harming non-target organisms (such as beneficial insects like bees and other soil biota), and posing risks to human health through the ingestion of crops containing fungicide residues.^18^

A recent study conducted by Lilai *et al*.^19^ in Tanzania demonstrated the effectiveness of two BCAs grown in commercially available media, *Trichoderma asperellum* (a fungus) and four *Bacillus* strains (*Bacillus subtilis* 4/5021 and *Bacillus velenzesis* strains 10/5140, 11/A+1, and 13/A+3), particularly in synergistic applications against cashew (*Anacardium occidentale* L.) wilt disease caused by the pathogenic fungus *Fusarium oxysporum*. However, reliance on synthetic media for BCA production has been deemed impractical for commercial use or application at the farm level, as these media are expensive and often result in low survival rates (shelf life) under field conditions. Therefore, the present study aimed to assess, determine, and optimize the most suitable organic substrates from five locally available options for the formulation of effective biofungicides targeting *F. oxysporum*, the causal agent of cashew fusarium wilt disease.

## 2. Materials and methods

### 2.1. Experimental design

The experiment was conducted under controlled conditions for two consecutive periods: June – December 2022 and January – June 2023, at the University of Dar es Salaam, Tanzania. *In vitro*, four bacterial strains, *B. subtilis* 4/5021 and *B. velenzesis* (strains 10/5140, 11/A+1, and 13/A+3), and the fungus *T. asperellum* were cultured separately on five locally available organic substrates: RH, rice bran (RB), hulled millets (HM), parboiled rice (PR), and a combination of all four (RH + RB + HM + PR) in a 1:1:1:1 ratio. Control treatments were also included. The *Bacillus* strains were combined synergistically, resulting in a total of 12 treatments: Two BCAs × five substrates + two control treatments. Synthetic growth media, Luria-Bertani liquid broth (LB broth) and potato dextrose broth (PDB), served as control treatments for bacterial and fungal growth, respectively. The experiment followed a completely randomized design with three replications, each consisting of a net plot of a single flask. The experiment was conducted at three different temperatures (4°C, 28°C, and 37°C) to assess growth performance and shelf-life stability across substrates.

### 2.2. Source of BCAs

The bacterial strains (*B. subtilis* 4/5021 and *B. velenzesis* strains 10/5140, 11/A+1, and 13/A+3) were obtained from the University of Dar es Salaam, Tanzania, which had previously received them from the University of Pretoria, South Africa. The fungal strain *T. asperellum* was sourced from the biofungicide Real IPM Tanzania Limited. Bacterial stock cultures were preserved in cryotubes at −20°C. Before use, *Bacillus* spores were cultured in LB broth (Difco^™^ LB Broth Miller, [Becton, Dickinson and Company] Biosciences, USA) at 37°C, and spores were harvested after 5 days by successive filtration using sterile Whatman filter paper number 1. *T. asperellum* was cultivated in potato dextrose agar (PDA) and collected after 7 days of incubation at 28°C.

### 2.3. Source and collection of locally available organic substrates

Five locally available organic substrates, as mentioned in Section 2.1, were used in the current study. Soybean cakes and molasses were also included as nutritional supplements to enrich the substrate bioformulation. The milling byproducts were obtained from local milling machines, whereas millet, rice grains, soybean cakes, and molasses were purchased from local cereal grain markets and animal/poultry feed stores in Tanzania.

### 2.4. Preparation of organic substrates for bioformulation development

Before further processing, cereal grains (HM) and rice byproducts were obtained after milling, where parboiled (partial-boiled) rice was initially soaked for 30 min and then steamed for the same duration before being dried. The locally obtained organic substrates were then blended into a fine powder using a heavy-duty professional blender (model number CJ-2020; Corolla; Japan; size 2.0 L). The nutritional contents of the substrates, specifically carbohydrates and proteins, were estimated colorimetrically after autoclaving at 121°C on a spectrophotometer. Ten grams of each blended substrate were transferred into 500 mL capacity volumetric flasks (five for *Bacillus* strains and five for *T. asperellum*), each containing distilled water. The substrates were sterilized at 121°C for 15 min at 1 atm, followed by filtration using a plastic mesh strainer to obtain a clear suspension. Before inoculation of the BCAs, the suspension was filtered again after 24 h.

### 2.5. Spore production and estimation

Conidia and endospore production of *T. asperellum* and the combined *Bacillus* strains in the substrate suspensions were estimated using the serial dilution method on PDA and plate count agar (PCA), respectively. Before serial dilution, the BCAs were re-isolated from all substrate-bioformulations. To prepare the serial dilutions, 1 mL of each substrate bioformulation was suspended in 9 mL of sterile distilled water, followed by vigorous shaking using a vortex mixer for 30 s. Twelve 10-mL dilution tubes were prepared for two substrate bioformulations (*Bacillus* combined strains and *T. asperellum*), with two control dilution tubes. Each bioformulation was serially diluted into dilution tubes labeled 10^2^, 10^4^, 10^6^, 10^7^, 10^8^, and 10^9^, each containing 1 mL of sterile distilled water as the diluent. The colony-forming units (CFU) per mL of substrate bioformulation were estimated using the following formula:


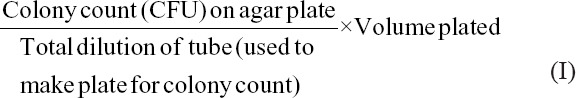


Where;


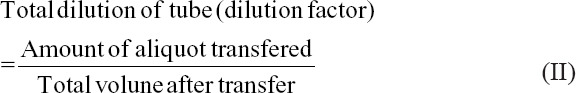


In this case, total volume after transfer = amount of aliquot transferred + amount of diluent already in tube.

The pour plate technique was used to obtain plates with a countable number of viable spores/endospores. A 0.1 mL (100 μL) aliquot of the serially diluted sample was plated onto agar plates (PDA and PCA). The plates were then incubated at 37°C for *Bacillus* strains and 28°C for *T. asperellum* until the colony growth was visibly evident for counting. The number of colonies was estimated visually and recorded as CFU per mL of substrate bioformulation, as described in literature.^2^ Colonies numbering between 30 and 300 in a plate were considered to be in the standard countable range, whereas fewer than 30 colonies were regarded too few to count, and more than 300 colonies were considered too many to count.^20^,^21^

### 2.6. Viability and shelf-life bioassay studies

Bioassay studies on shelf life and spore viability were conducted by storing substrate bioformulation in sterile 250 – 500 mL volumetric flasks, sealed with sterile cotton wool, and wrapped in aluminum foil. These flasks were kept at 28°C and 37°C, while sterile plastic screw-cap bottles were used for storage in the refrigerator at 4°C. The shelf life of conidia and endospores was assessed after 15 days and monitored monthly for 6 months in each season. The shelf life of conidia and endospores was determined as described in aforementioned equations (I) and (II). For each assessment, 0.1 mL (100 μL) of the suspension was taken and suspended in 9 mL of sterile distilled water. Spore production and viability were estimated visually and recorded as CFU per mL of substrate bioformulation. CFU was initially calculated to estimate the number of viable, colonogenic cells in CFU/mL. The CFU per mL indicates the number of cells/spores that remained viable (alive) and capable of further multiplication.

### 2.7. Statistical analysis

Repeated measures multivariate analysis of variance was used to study the influence of substrates on the proliferation of BCAs across three different temperatures and their viability over time (months), as compared with the control treatments (LB broth/PDB) at *p* ≤ 0.05.

A one-way analysis of variance was performed to examine the population density of the two BCAs (*Bacillus* strains and *T. asperellum*) across the organic-based substrates over two consecutive periods (2022 and 2023), and the Tukey test was conducted for means separation. Before analysis, all data (CFU/mL) were normalized by log transformation. All analyses were performed using GenStat discovery software (version 15.1, release PL 23.1, VSN International [VSNi], UK).

## 3. Results

### 3.1. Carbohydrate and protein contents in the prepared substrates

The colorimetric analysis of the substrates revealed that the protein and carbohydrate contents ranged from 13.4% to 19.8% and from 76.3% to 82.7%, respectively (Table 1). The highest protein content was recorded in RB, followed by RH and the combined substrates, whereas the carbohydrate content was found to be high in RB, followed by combined substrates and HM.

**Table 1:** Carbohydrate and protein contents of the substrates

Substrates	Protein (%)	Carbohydrate (%)
Rice husk	18.7	76.3
Hulled millet	17.6	80.2
Parboiled rice	13.4	79.4
Rice bran	19.8	82.7
Combined substrates	17.4	80.6

### 3.2. Colonization and sporulation of the BCAs

The proliferation of BCAs on the selected organic substrates was visually observed and subsequently confirmed through re-isolation on nutrient agar and PDA. The cultural and microscopic results of the re-isolation showed the presence of similar BCAs, including *Bacillus* strains and *T. asperellum*, on the colonized substrates. The results indicated that, after 15 days of incubation at 28 ± 2°C and 37°C, all substrates were colonized to varying extents. The colonization intensity of *Bacillus* strains and *T. asperellum* at 28 ± 2°C on RH was higher than at 37°C (growth at 28 ± 2°C >37°C). Conversely, in RB, growth was higher at 37 °C than at 28 ± 2°C. The colonization intensity and population density of the formulated biofungicides of *Bacillus* strains and *T. asperellum* varied among the substrates over time (months) during both seasons (2022 and 2023), as presented in Tables 2 and 3. In general, the population density ranged from 0.0 (not available [n/a]) to 2.1 × 10^7^ ± 4.9 × 10^5^ CFUs/mL at 28 ± 2°C and 0.0 [n/a] to 3.5 × 10^8^ ± 7.4 × 10^7^ CFUs/mL at 37°C in 2022. In 2023, the population densities ranged from 0.0 [n/a] to 1.0 × 10^7^ ± 0.3 × 10^5^ CFUs/mL at 28 ± 2°C and from 0.0 [n/a] to 8.3 × 10^7^ ± 6.2 × 10^6^ CFUs/mL at 37°C.

The colonization intensities of *T. asperellum* and *Bacillus* strains in RH and combined substrates were higher at 28 ± 2°C in both seasons. The final population densities of *T. asperellum* and *Bacillus* strains in RH and combined substrates for both seasons are listed in Tables 2 and 3.

**Table 2:** Colonization intensity and population density of *Bacillus* strains and *Trichoderma asperellum* in different substrate bioformulations during season 2022 (experimental run 1)

Biological control agents	Growth substrates	Colonization intensity		Population density (CFU/mL of formulated biofungicides)				
	
		At 28±2°C	At 37°C	At 28±2°C		At 37°C		At 4°C
		
				Initial PD	Final PD	Initial PD	Final PD	Final PD
*Bacillus* strains	Rice husk	+++	++	5.3×10^4^±3.4×10^2b^	2.5×10^3^±2.3×10^2d^	5.9×10^6^±1.9×10^5a^	2.3×10^7^±5.4×10^5b^	6.0×10^7^±5.1×10^5c^
	Hulled millet	+	+	4.1×10^3^±7.4×10^2c^	1.0×10^1^±0.2×10^1f^	1.7×10^3^±2.0×10^2c^	0.7×10^1^±0.2×10^1d^	3.6×10^5^±1.7×10^4e^
	Parboiled rice	++	+++	1.4×10^5^±1.9×10^4a^	1.2×10^3^±1.1×10^1d^	1.2×10^2^±1.0×10^1d^	1.0×10^1^±0.2×10^1d^	3.4×10^7^±2.3×10^6c^
	Rice bran	++	+++	4.2×10^5^±8.6×10^4a^	1.1×10^2^±1.8×10^1e^	7.2×10^4^±3.2×10^3b^	3.6×10^5^±2.1×10^4c^	6.5×10^9^±5.9×10^7a^
	Combined substrates	+++	+++	3.0×10^5^±9.7×10^3a^	2.1×10^7^±4.9×10^5a^	8.3×10^6^±2.6×10^5a^	3.5×10^8^±7.4×10^7a^	7.7×10^8^±3.2×10^7b^
	LB broth	++	+++	3.0×10^5^±9.4×10^3a^	1.3×10^3^±3.7×10^5d^	8.1×10^6^±2.6×10^5a^	5.3×10^5^±2.3×10^4c^	4.4×10^8^±2.9×10^7b^
*Trichoderma asperellum*	Rice husk	+++	++	1.5×10^4^±2.3×10^3b^	1.1×10^4^±4.5×10^3c^	1.3×10^2^±1.0×10^1d^	0.2×10^1^±0.1×10^1d^	3.5×10^9^±2.9×10^8a^
	Hulled millet	+++	++	4.2×10^4^±3.9×10^3b^	2.0×10^2^±0.1×10^1ef^	1.1×10^4^±8.3×10^3b^	n/a	2.5×10^6^±7.4×10^5d^
	Parboiled rice	+	+	3.0×10^4^±6.3×10^2b^	n/a	1.4×10^3^±1.4×10^2c^	n/a	2.7×10^6^±2.6×10^5d^
	Rice bran	+	+	2.1×10^4^±2.7×10^3b^	1.0×10^2^±1.3×10^1e^	1.0×10^4^±3.7×10^3b^	n/a	1.8×10^8^±4.7×10^7bc^
	Combined substrates	+++	++	2.9×10^5^±3.3×10^4a^	2.4×10^6^±7.3×10^4b^	2.7×10^3^±2.3×10^2c^	1.0×10^1^±0.1×10^1d^	2.8×10^7^±1.8×10^6c^
	PDB	+++	++	2.6×10^5^±3.7×10^4a^	1.8×10^6^±7.3×10^4b^	2.4×10^3^±2.3×10^2c^	n/a	2.6×10^8^±4.3×10^7bc^

Notes: Colonization intensity: + = low; ++ = medium; +++ = high; n/a=not available (0 CFU/mL). Initial population densities were enumerated after 15 days of incubation of the growth substrates at 28±2℃ and 37℃. LB broth and PDB served as positive controls for *Bacillus* strains and *T. asperellum*. The final population density of the biological control agents was recorded after 6 months in 2022 (first run test). The initial population density at 4℃ was not included, as only the final population density was of interest for shelf-life studies. Data are presented as mean values±standard error from three replicates. Means in each column followed by the same superscript letter are not significantly (*p*>0.05) different according to the Tukey test. Abbreviations: CFU: Colony-forming units; LB: Luria–Bertani; PD: Population density; PDB: Potato dextrose broth.

**Table 3:** Colonization intensity and population density of *Bacillus* strains and *Trichoderma asperellum* in different substrate bioformulations during season 2023 (experimental run 2)

Biological control agents	Growth substrates	Colonization intensity		Population density (CFU/mL of formulated biofungicides)				
	
		At 28±2°C	At 37°C	At 28±2°C		At 37°C		At 4°C
		
				Initial PD	Final PD	Initial PD	Final PD	Final PD
*Bacillus* strains	Rice husk	+++	++	5.1×10^4^±1.2×10^3b^	2.7×10^3^±1.1×10^2d^	5.4×10^6^±4.2×10^5a^	5.7×10^7^±1.1×10^6a^	5.2×10^9^±4.4×10^7a^
	Hulled millet	+	+	3.9×10^3^±5.3×10^2c^	0.3×10^4^±0.3×10^3c^	1.5×10^3^±1.0×10^2c^	0.3×10^1^±0.2×10^1d^	3.5×10^6^±2.1×10^4d^
	Parboiled rice	++	+++	1.4×10^5^±1.2×10^4a^	1.2×10^3^±1.2×10^2d^	1.1×10^2^±1.0×10^1d^	1.0×10^1^±0.1×10^1c^	3.6×10^7^±4.5×10^6c^
	Rice bran	++	+++	4.4×10^5^±3.9×10^4a^	0.1×10^2^±0.3×10^1e^	7.3×10^4^±3.8×10^3b^	3.3×10^5^±1.6×10^4b^	3.6×10^8^±5.3×10^6b^
	Combined Substrates	+++	+++	3.7×10^5^±3.5×10^4a^	1.0×10^7^±0.3×10^5a^	8.1×10^6^±1.8×10^5a^	8.3×10^7^±6.2×10^6a^	5.8×10^9^±2.8×10^8a^
	LB Broth			3.2×10^5^±3.3×10^4a^	1.1×10^4^±1.2×10^3c^	7.8×10^6^±1.6×10^5a^	3.9×10^5^±1.7×10^4b^	4.8×10^7^±1.6×10^7c^
*T. asperellum*	Rice husk	+++	++	1.3×10^4^±1.7×10^2b^	0.9×10^4^±0.2×10^2c^	1.3×10^2^±1.7×10^1c^	0.2×10^1^±0.1×10^1d^	3.3×10^6^±1.5×10^4d^
	Hulled millet	+++	++	4.0×10^4^±2.8×10^3b^	0.2×10^4^±0.6×10^3c^	1.3×10^4^±1.1×10^2b^	0.3×10^1^±0.1×10^1d^	2.7×10^7^±3.2×10^6c^
	Parboiled rice	+	+	3.2×10^4^±4.1×10^2b^	n/a	1.3×10^3^±1.0×10^2c^	n/a	2.7×10^5^±1.8×10^4e^
	Rice bran	+	+	2.5×10^4^±5.3×10^3b^	1.5×10^2^±1.1×10^1e^	1.0×10^4^±1.0×10^2b^	n/a	2.5×10^7^±5.7×10^6c^
	Combined substrates	+++	++	2.0×10^5^±6.1×10^4a^	2.1×10^6^±3.6×10^4b^	2.3×10^3^±1.7×10^2c^	1.0×10^1^±0.3×10^1c^	2.3×10^7^±1.1×10^6c^
	PDB			2.9×10^5^±5.3×10^4a^	1.1×10^6^±2.7×10^4b^	2.5×10^3^±1.4×10^2c^	n/a	1.9×10^7^±4.3×10^7c^

Notes: Colonization intensity: + = low; ++ = medium; +++ = high; n/a=not available (0 CFU/mL). Initial population densities were enumerated after 15 days of incubation of the growth substrates at 28±2℃ and 37℃. LB broth and PDB were used as positive controls for *Bacillus* strains and *T. asperellum*. The final population density of the biological control agents was recorded after 6 months in 2023 (second run test). The initial population density at 4℃ was not included, as only the final population density was of interest for shelf-life studies. Data are presented as mean values±standard error from three replicates. Means in each column followed by the same superscript letter are not significantly (*p*>0.05) different according to the Tukey test. Abbreviations: CFU: Colony-forming units; LB: Luria-Bertani;

PD: Population density; PDB: Potato dextrose broth.

However, the colonization intensity varied significantly between *T. asperellum* and *Bacillus* strains at 37°C. For example, after 6 months of incubation in each season (2022 and 2023), the population densities of *Bacillus* strains in combined substrates remained high, being 3.5 × 10^8^ ± 7.4 × 10^7^ and 8.3 × 10^7^ ± 6.2 × 10^6^ CFUs/mL, respectively. In contrast, the population densities of *T. asperellum* were much lower, being 1.0 × 10^1^ ± 0.1 × 10^1^ and 1.0 × 10^1^ ± 0.3 × 10^1^ CFUs/mL, respectively. Similarly, in both seasons, the level of endospore formation and conidiation also varied greatly in RH, with *Bacillus* strains remaining high, being 2.3 × 10^7^ ± 5.4 × 10^5^ and 5.7 × 10^7^ ± 1.1 × 10^6^ CFUs/mL, respectively, compared to *T. asperellum*, which registered a lower value of 0.2 × 10^1^ ± 0.1 × 10^1^ CFUs/mL in each season. Other substrates showed minimal colonization, with lower population densities compared to the above-mentioned substrates (Tables 2 and 3).

### 3.3. Viability and shelf-life bioassay studies

The results on viability demonstrated significant variation (*p* ≤ 0.05) between the viability of BCAs and incubation temperatures over time (Table 4).

**Table 4:** Repeated measures multivariate analysis of variance showing the influence of organic substrates on the proliferation of two biological control agents at three temperatures (room temperature, incubator, and refrigerator temperatures)

Term	d.f.	Wilk’s lambda	Rao F	n.d.f.	d.d.f.	*F* prob.	Pillai–Bartlett trace	Roy’s maximum root test	Lawley–Hotelling trace
Temperature	2	0.1563	3.06	22	44	0.001	1.1644	0.7172	3.345
Covariate	1	0.6913	0.89	11	22	0.561	0.3087	0.3087	0.447

Notes: Covariate=biofungicides; Room temperature=28±2°C; Incubator temperature=37°C; Refrigerator temperature=4°C. Abbreviations: d.d.f.: Degrees of freedom for the denominator; d.f.: Degrees of freedom; n.d.f.: Degrees of freedom for the numerator; prob: Probability; Rao F: Rao’s F Statistic (Statistical measure used in multivariate analysis to test hypotheses about the differences between groups).

**Figure 1:**
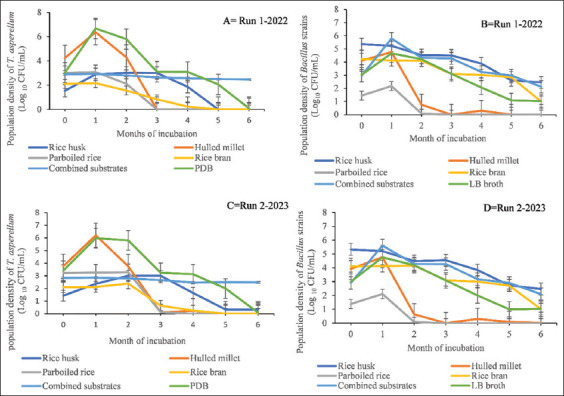
Viability of *Trichoderma asperellum* (A and C) and *Bacillus strains* (B and D) in five organic substrates after 6 months of incubation at room temperature (28 ± 2°C) during the 2022 and 2023 seasons. Initial population densities (month 0) were enumerated after 15 days of incubation of the growth substrates at 28 ± 2°C. Bars in the line graphs represent the standard errors of the mean.

The population density of *T. asperellum* increased exponentially between days 15 and 30 at 28 ± 2°C in both seasons. Notably, the population began to decrease after 1 month, except in RH, where it continued to increase until 2 months before exhibiting a steady decline, and in the combined substrates, where a gradual decline was observed (Figure 1A and 1C). Similarly, the population density of *Bacillu*s strains began to decline after 1 month, while the population density in RH, combined substrates, and RB exceeded that of LB broth, which acted as a control treatment (Figure 1B and 1D). In contrast, population densities in PR and HM declined more rapidly as compared to control treatments during the same period. In addition, both *T. asperellum* and *Bacillus* strains maintained stable population densities in combined substrates and RB throughout the entire experimental period (run 1 – 2022 and run 2 – 2023), although they dropped gradually. An exception was *Bacillus* strains in RH, which remained stable for the same period.

**Figure 2:**
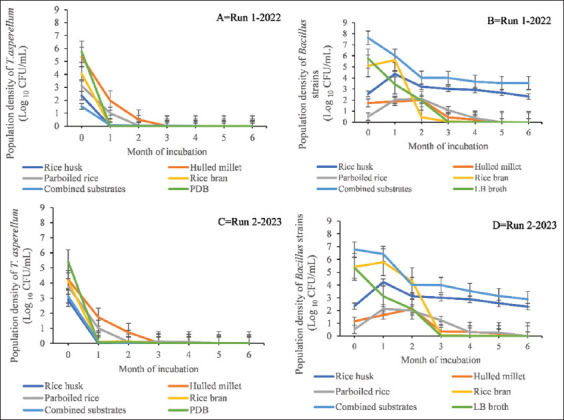
Viability of *Trichoderma asperellum* (A and C) and *Bacillus strains* (B and D) in five organic substrates after 6 months of incubation at 37°C during the 2022 and 2023 seasons. Initial population densities (month 0) were enumerated after 15 days of incubation of the growth substrates at 37°C. Bars in the line graphs represent the standard errors of the mean.

At 37°C, the population density of *T. asperellum* in all substrates, including the control treatments, was lower than that of *Bacillus* strains, and began to decline after 5 days of incubation (Figure 2A and 2C). The final population density of *T. asperellum* ranged from 0.0 to 1.0 × 10^1^ ± 0.3 × 10^1^ CFUs/mL in both seasons, whereas *Bacillus* strains ranged from 0.7 × 10^1^ ± 0.2 × 10^1^ to 3.5 × 10^8^ ± 7.4 × 10^7^ CFUs/mL in 2022, and from 0.3 × 10^1^ ± 0.2 × 10^1^ to 8.3 × 10^7^ ± 6.2 × 10^6^ CFUs/mL in 2023 (Figure 2, Tables 2 and 3).

**Figure 3:**
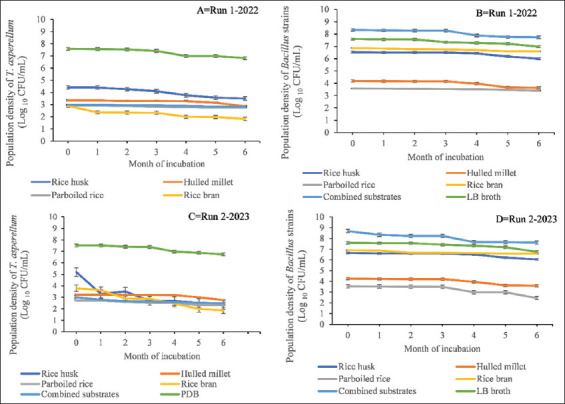
Viability of *Trichoderma asperellum* (A and C) and *Bacillus strains* (B and D) in five organic substrates after 6 months of incubation at refrigerator temperature (4°C) during the 2022 and 2023 seasons. Initial population densities (month 0) were enumerated after 15 days of incubation of the growth substrates at 4°C. Bars in the line graphs represent the standard errors of the mean.

Overall, combined substrates and RH maintained the viability of *T. asperellum* and *Bacillus* strains at 28 ± 2°C and 37°C, respectively, throughout both experimental runs (run 1 – 2022 and run 2 – 2023). In contrast, the populations of both BCAs in all substrates remained stable when refrigerated at 4°C throughout the experimental periods in both runs (2022 and 2023), with no significant variability found among them (Figure 3). For instance, the viability of *T. asperellum* in RB gradually decreased from month 0 (2.8 × 10^4^ ± 1.1 × 10^3^ CFUs/mL) up to month 6 (1.8 × 10^4^ ± 2.3 × 10^2^ CFU/mL), while the viability of *Bacillus* strains in the same substrate dropped from 6.7 × 10^6^ ± 2.1 × 10^5^ to 6.6 × 10^6^ ± 2.6 × 10^3^ CFUs/mL in experimental run 1 – 2022 (Figure 3). Similarly, in experimental run 2 – 2023, the viability of *T. asperellum* dropped from month 0 (3.8 × 10^7^ ± 1.3 × 10^6^ CFU/mL) to month 6 (1.9 × 10^7^ ± 5.7 × 10^6^ CFU/mL), while *Bacillus* strains declined from month 0 (6.9 × 10^8^ ± 1.8 × 10^6^ CFU/mL) to month 6 (6.5 × 10^8^ ± 3.3 × 10^6^ CFUs/mL).

## 4. Discussion

### 4.1. Proliferation of BCAs on organic substrates

#### 4.1.1. Carbohydrate and protein contents in organic substrates

The development of bacterial- and/or fungal-based bioformulations depends on several factors, including the availability of adequate nutrients in the substrate, population density of microbes, temperature, pH, moisture, and other bioactive compounds.^22^ The growth and viability of BCAs require various nutrients, such as proteins, carbohydrates, phosphorus, and other essential trace bioactive elements. Carbohydrates and proteins serve as the primary sources of energy and amino acids, which are crucial for microbial growth and proliferation. A deficiency or low level of these nutrients in a bioformulation can present a critical challenge to the development of biofungicides, potentially resulting in the short-term viability (shelf life) of microbial products. For instance, studies by Abbasi *et al*.^23^ have demonstrated that rice agro-wastes and chickpeas are rich in carbohydrates, proteins, minerals, vitamins, and other bioactive compounds vital for bacterial proliferation and the production of bioactive metabolites. In addition, the rice-based organo-substrates contain vitamin B1 (thiamine), a coenzyme responsible for carbohydrate and protein metabolism, which enhances the proliferation of BCAs grown in the thiamine-containing substrates.^24^ Our findings also revealed adequate levels of carbohydrates and proteins in the studied substrates, suggesting that these substrates are rich in vitamin B1 (thiamine), which serves as a critical source of energy and amino acids, fundamental for the growth and proliferation of microbes in their natural environments. These results are consistent with Devi *et al*.^25^ and Nnadiukwu *et al.*,^26^ who reported that RB contains 11 – 17% protein and RH contains 37.04 g/100 g of carbohydrates with 1.85 g/100 g of crude protein. In addition, Amagliani *et al*.^27^ and Wisetkomolmat *et al*.^28^ reported that RB contains more nutrients and vitamins than other rice milling byproducts, noting a protein content of about 11 – 15% and 78% carbohydrate.

#### 4.1.2. Colonization and sporulation of BCAs

The level of endospore formation and conidiation varied among the bioformulations of the substrates (Tables 2 and 3). The diversity of nutritional contents in the organic substrates may have contributed to the variation in endospore and conidia formation,^1^ which aligns with our current findings that revealed adequate levels of proteins and carbohydrates across the substrates (Table 1). Many members of the genera *Bacillus* and *Trichoderma* possess cellulolytic properties and are potential lignocellulose degraders. Our research primarily utilized rice-based materials as sources of growth substrates. According to Sala *et al*.^7^ and Dobrzy’nski *et al.*,^29^ rice-based agro-wastes, such as RH and rice straws, are rich in cellulose, hemicellulose, and lignin, making them ideal sources of nutrients for the growth and proliferation of *Bacillus* and *Trichoderma* strains. These genera are capable of hydrolyzing cellulose, hemicellulose, and lignin through the action of cellulase and hemicellulase enzymes. Ahmed *et al*.^30^ identified 28°C as the optimal temperature for *Trichoderma harzianum* to produce cellulase enzymes, a finding consistent with our study. Our results showed a higher colonization intensity of BCAs at 28 ± 2°C compared to 37°C when grown in RH, while colonization was higher at 37°C than at 28 ± 2°C in RB. However, Naeimi *et al*.^2^ reported that the extent of colonization intensity does not necessarily correlate with conidiation in each substrate. This is also reflected in our findings, where the colonization of both *T. asperellum* and *Bacillus* strains was higher in HM, yet the estimated CFU was smaller compared to other substrates (Tables 2 and 3). The formation of endospores and conidia is influenced by various factors, including the formulation method, temperature, bioactive ingredients, pH, and substrate particle size, as well as high protein and fat content.^2^,^3^,^8^ In addition, some substrates favor vegetative growth over the production of endospores and conidia.^31^

At 6 months, the population densities (CFU) of both *T. asperellum* and *Bacillus strains* were higher when incubated at 28 ± 2°C in combined substrates during each season. The proteins, carbohydrates, and other bioactive compounds present in these substrates serve as key nutrients, supporting the growth and proliferation of BCAs in the soil ecosystem.^32^ This benefit could have contributed to the high proliferation and endospore/conidiation formation observed in our study. Similarly, Ravimannan *et al*.^9^ used parboiled longgrain white rice and HM as substrates for the growth of *Bacillus pumilus* and *Streptomyces griseus*, finding their carbohydrate and protein contents to be 77.8% and 8.9%, and 71.6% and 10.5% for PR and HM, respectively. Moreover, some substrates exhibited lower population densities (CFU), while others showed no population at 6 months in each season (Figures 1 and 2, and Tables 2 and 3). This observation could be ascribed to competition among the BCAs for nutrients, as noted by Lin and Ho.^33^

### 4.2. Viability and shelf-life bioassay studies

The exponential growth observed in *T. asperellum* between days 15 and 30 at a temperature of 28 ± 2°C may be attributed to the utilization of organic substrates, which serve as important microbial food sources. For example, Cumagun and Naeimi *et al*.^2^,^34^ highlighted the potential of organic food bases to enhance the proliferation of BCAs compared to inorganic food bases. Thus, the nutritional contents and other bioactive compounds in these substrates likely contributed to the rapid growth of BCAs (Figure 1A and 1C). Nevertheless, at certain points during growth, as indicated by the curves, a decline occurred due to nutrient depletion and the production of secondary metabolites that hindered further growth.^35^,^36^ Notably, the final population density of *T. asperellum* in combined substrates and RB remained higher than in other substrates at 28 ± 2°C. The high protein and carbohydrate content (Table 1), in combination with the temperature effect, may have supported both the growth and conidiation of *T. asperellum*. Similarly, studies by Lee *et al.*, Carro-Huerga *et al.*, and Mustafa *et al*.^1^,^37^,^38^ reported that organic substrates rich in carbohydrates and proteins, as well as temperature, influence fungal growth, particularly in members of the genus *Trichoderma*. These studies indicated that most fungi grow well within a broad temperature range of 25 – 33°C, with an optimum temperature of 30°C. Consistent with this, the United States Department of Agriculture^39^ reported that the nutritional contents of HM include 71.6% carbohydrates and 10.5% proteins, which are sufficient to support the growth and multiplication of BCAs. In contrast, the population densities of *Bacillus* strains at 28 ± 2°C were relatively low, except in the combined substrates, where the final calculated CFU was higher than those in other substrates. This variation in endospore formation may be attributed to the temperature effect, as some studies suggest that the optimal growth temperatures for *Bacillus* species range from 35°C to 38°C, with a growth range between 25°C and 40°C.^40^ Despite the differences in population dynamics displayed between the two BCAs at 28 ± 2°C, combined substrates, RB, and RH supported the growth of both BCAs throughout the entire 6-month experimental period in each season.

At 37°C, a variation in viability among the substrates was observed for both BCAs. Despite these variations, *T. asperellum* generally exhibited considerably lower CFU than *Bacillus* strains in most substrates over the incubation periods (Figure 2A and 2C). For instance, there were no viable cells of *T. asperellum* in any substrate at the end of the incubation period in both seasons, except in RB and combined substrates, which still contained viable cells up to the 3^rd^ month (Figure 2A and 2C). Meanwhile, combined substrates and RH, followed by RB, maintained viable *Bacillus* cells throughout the 6-month period in both seasons. The stability in the viability of *Bacillus* strains can likely be attributed to the high nutrient content, favorable growth temperature, and the presence of thiamine (vitamin B1), an important coenzyme found in the organic substrates. The low CFU of *T. asperellum* in the substrates may be due to the temperature effect, as the optimal growth for most fungi, including *T. asperellum*, is typically within the range of 25 – 33°C. At higher temperatures, such as 37°C, enzyme activities responsible for metabolic processes may become less efficient, reducing growth and reproduction. In addition, high temperatures can destabilize cell membranes, leading to cellular damage.

Overall, all five substrates supported the growth of *T. asperellum* and *Bacillus* strains at both 28 ± 2°C and 37°C. However, the combined substrate proved to be the most effective, supporting the colonization and proliferation of both BCAs throughout the entire 6-month period in each season, followed by RH and RB. Nevertheless, the viability and population densities of *T. asperellum* and *Bacillus* strains in all substrate-based bioformulations were less affected at refrigeration temperature (4°C) than at room or incubator temperatures (Figure 3). These findings align with other studies that have shown the effects of high temperatures over protracted incubation periods, which ultimately impact cell viability.^41^-^43^

## 5. Conclusion

The results demonstrated that all five tested substrates were successfully colonized by the BCAs, with RB exhibiting the highest colonization rate, followed by RH and the combined substrates. The shelf-life and viability evaluation indicated that the endospore formation and conidiation capabilities of the BCAs varied over time and across the substrates. Population densities of the BCAs decreased before the 6^th^ month in each season, except for those in combined substrates, RH, and RB, which maintained relatively higher densities. However, when stored at 4°C in a refrigerator, the CFU of the BCAs remained high. Based on these findings, combined substrates, RH, and RB are recommended as suitable growth media for the large-scale production of BCAs *T. asperellum* and *Bacillus* strains. This research, therefore, offers a cost-effective solution for utilizing locally available organic substrates in developing countries, enabling the implementation of biotechnological strategies to manage cashew fusarium wilt disease.

## Data Availability

The data supporting the findings of this study are available within the article.
